# Straw and Green Manure Return Can Improve Soil Fertility and Rice Yield in Long-Term Cultivation Paddy Fields with High Initial Organic Matter Content

**DOI:** 10.3390/plants14131967

**Published:** 2025-06-27

**Authors:** Hailin Zhang, Long Chen, Yongsheng Wang, Mengyi Xu, Weiwen Qiu, Wei Liu, Tingyu Wang, Shenglong Li, Yuanhang Fei, Muxing Liu, Hanjiang Nie, Qi Li, Xin Ni, Jun Yi

**Affiliations:** 1 Hubei Province Key Laboratory for Geographical Process Analysis and Simulation, Central China Normal University, Wuhan 430079, China; 2 Institute of Geographic Sciences and Natural Resources Research, Chinese Academy of Sciences, Beijing 100101, China; 3 The New Zealand Institute for Plant and Food Research Limited, Private Bag 3230, Hamilton 3240, New Zealand; 4 Engineering Research Center of Photoenergy Utilization for Pollution Control and Carbon Reduction, Ministry of Education, Wuhan 430079, China

**Keywords:** straw return, green manure, soil organic matter, rice yield, high-fertility soil

## Abstract

Returning straw and green manure to the field is a vital agronomic practice for improving crop yields and ensuring food security. However, the existing research primarily focuses on drylands and low-fertility paddy fields. A systematic discussion of the yield-increasing mechanisms and soil response patterns of medium- and long-term organic fertilization in subtropical, high-organic-matter paddy fields is lacking. This study conducted a six-year field experiment (2019–2024) in a typical high-fertility rice production area, where the initial organic matter content of the 0–20 cm topsoil layer was 44.56 g kg^−1^. Four treatments were established: PK (no nitrogen, only phosphorus and potassium fertilizer), NPK (conventional nitrogen, phosphorus, and potassium fertilizer), NPKM (NPK + full-amount winter milk vetch return), and NPKS (NPK + full-amount rice straw return). We collected 0–20 cm topsoil samples during key rice growth stages to monitor the dynamic changes in nitrate and ammonium nitrogen. The rice SPAD, LAI, plant height, and tiller number were also measured during the growth period. After the six-year rice harvest, we determined the properties of the topsoil, including its organic matter, pH, total nitrogen, phosphorus, potassium, available phosphorus and potassium, and alkali hydrolyzable nitrogen. The results showed that, compared to NPK, the organic matter content of the topsoil (0–20 cm) increased by 6.36% and 5.16% (annual average increase of 1.06% and 0.86%, lower than in low-fertility areas) in the NPKS and NPKM treatments, respectively; the total nitrogen, phosphorus, and potassium content increased by 16.59%, 8.81%, and 10.37% (NPKS) and 6.70%, 5.12%, and 11.62% (NPKM), respectively; the available phosphorus content increased by 21.87% and 8.42%, respectively; the available potassium content increased by 47.38% and 11.56%, respectively; and the alkali hydrolyzable nitrogen content increased by 3.24% and 2.34%, respectively. However, the pH decreased by 0.07 in the NPKS treatment while it increased by 0.17 in the NPKM treatment, respectively, compared to the PK treatment. NPKS and NPKM improved key rice growth indicators such as the SPAD, LAI, plant height, and tillering. In particular, the tillering of the NPKS treatment showed a sustained advantage at maturity, increasing by up to 13.64% compared to NPK, which also led to an increase in the effective panicle number. Compared to NPK, NPKS and NPKM increased the average yield by 9.52% and 8.83% over the six years, respectively, with NPKM having the highest yield in the first three years (2019–2021) and NPKS having the highest yield from the fourth year (2022–2024) onwards. These results confirm that inputting organic materials such as straw and green manure can improve soil fertility and rice productivity, even in rice systems with high organic matter levels. Future research should prioritize the long-term monitoring of carbon and nitrogen cycle dynamics and greenhouse gas emissions to comprehensively assess these practices’ sustainability.

## 1. Introduction

Rice, a staple crop for over 3 billion people worldwide, is strategically significant for ensuring global food security [1]. However, to guarantee yields, intensive land use and the extensive application of single-type fertilizers, which lead to soil degradation, are common problems facing global rice production [2,3,4]. Research indicates that widespread practices in global paddies, such as excessive nitrogen fertilizer application, continuous monoculture cropping, and excessive mechanical tillage, have led to secondary obstacles like soil acidification, nutrient imbalance, and structural compaction, significantly limiting rice production potential [5]. Against this backdrop, how to achieve both high and stable rice yields and the synergistic improvement of soil fertility has become a core issue that urgently requires solutions for sustainable agricultural development to be achieved [6].

In recent years, researchers have developed multiple technical approaches focused on soil conservation and fertility improvement, including the return of organic materials (straw, manure) to the field, green manure cover cropping, and conservation tillage systems [7,8,9,10,11,12]. Numerous studies have confirmed that these measures significantly enhance the productivity and ecological stability of rice paddy systems by increasing their soil organic matter content, improving their aggregate structure, and increasing their microbial activity [13,14]. Among these, straw return and winter green manure are widely regarded as having potential for large-scale promotion due to their dual advantages of economic viability and operational feasibility.

The application of straw and green manure as exogenous organic matter amendments has been extensively studied and practiced worldwide; however, the conclusions regarding their impact on rice yield remain inconsistent. Two field experiments showed that straw return decreased the rice yield by 5.1–6.7% compared to no straw return [15,16]. A meta-analysis indicated that, in northern China, non-leguminous green manure reduced the wheat yield by 7.2% [17]. Various factors, including initial soil conditions, the climate, and management practices, influence the effect of returning straw and green manure to the field. Current research has primarily focused on low-fertility systems, and studies on the impact of straw and green manure input on the soil and crop yield in regions with high base fertility are relatively scarce.

Based on the soil carbon saturation theory, organic carbon pools have a defined upper limit for carbon input at a steady state. As the amount of organic carbon approaches the regional background threshold, its sequestration efficiency for exogenous carbon will decay exponentially [18,19]. High soil carbon and nitrogen stocks may significantly inhibit the sequestration efficiency of newly added organic carbon and nitrogen, and may thereby hinder crop yield increases. This phenomenon raises a significant practical question: in these paddy fields with high soil organic carbon (SOC) content (approaching carbon saturation), can the practices of straw return and winter green manure still improve soil fertility and, consequently, continue to increase rice yield?

The objectives of this study were as follows: (1) to evaluate whether straw return and winter green manure can consistently improve rice growth and increase grain yield; and (2) to determine whether soil fertility indicators will undergo significant changes with the input of organic matter such as straw and green manure.

## 2. Materials and Methods

### 2.1. Study Site

**Figure 1:**
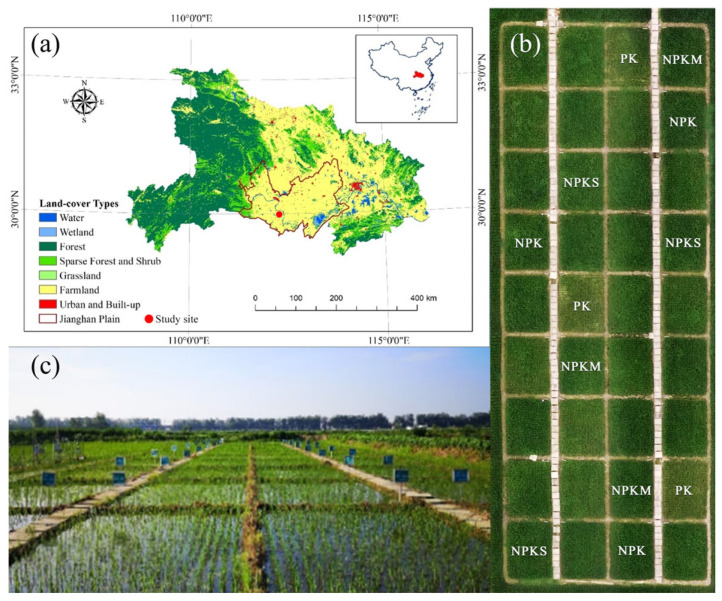
Geographical location of the study site (**a**), aerial photography distribution of different treatment plots (**b**), and panoramic map of the study site (**c**). Note: PK (no nitrogen, only phosphorus and potassium fertilizer), NPK (conventional nitrogen, phosphorus, and potassium fertilizer), NPKM (NPK + full-amount winter milk vetch return), and NPKS (NPK + full-amount rice straw return).

The study area was located in the south-center of Jianghan Plain (Figure 1a, 112°34′ E, 29°28′ N), which has a subtropical monsoon climate, an average annual temperature of 16 °C, and average annual rainfall and potential evaporation that range from 1000 to 1400 mm and from 1300 to 1800 mm, respectively, and these are primarily concentrated in the hotter months from April to September. Rice is the main food crop in the region and has been cultivated for thousands of years. The soil types in the experimental area are primarily paddy soils of the potential and subsurface water-retaining types with soil textures of loam and clay loam. The organic matter content of topsoil (0–20 cm) was 44.56 g kg^−1^ at the beginning of the experiment.

### 2.2. Experimental Design

In October 2017, a whole flat old rice field (2200 m^2^) with a rice cultivation history of over 100 years was selected as the experimental plot, which was divided into 9 rows and 4 columns, totaling 36 experimental plots, each with an area of 27 m^2^ (Figure 1b). Ridges were set between the plots, and impermeable plastic films were used to separate them to prevent water and fertilizer movement between plots. Twelve of these plots were randomly selected to conduct these treatments with three replications each (Figure 1b,c). The treatments included PK (no nitrogen, only phosphorus, and potassium fertilizer), NPK (conventional nitrogen, phosphorus, and potassium fertilizer), NPKM (NPK + full-amount winter milk vetch return), and NPKS (NPK + full-amount rice straw return). Except for PK, which did not apply nitrogen, the nitrogen fertilizer application rate for the remaining treatments was 225 kg N ha^−1^.

The research period was from 2019 to 2024, and the rice variety selection was consistent with local practices. According to the traditional habits of local farmers planting single-season mid-season rice, the fields were flooded and rotary tilled before planting. In 2019, rice was established using a broadcasting method; in other years, transplanting was used. Both transplanting and harvesting were carried out manually. The number of rice seedlings transplanted per plot was approximately 19.4 holes per square meter, with a seedling-raising period of 30 days and a whole-growth period of roughly 120~130 days. The nitrogen fertilizer applied to the plots was urea. The rice broadcast in 2019 was applied in 4 split applications, including basal fertilizer (30%), tillering fertilizer (35%), panicle fertilizer (25%), and grain-filling fertilizer (10%); the rice transplanted in 2020–2024 was applied in three split applications of basal fertilizer, tillering fertilizer, and panicle fertilizer, with application ratios of 35%, 35%, and 30%, respectively. Phosphorus and potassium fertilizers were applied as P_2_O_5_ and K_2_O, respectively, and were only applied once as basal fertilizer, with application rates of 40 kg and 60 kg ha^−1^, respectively. Except during the late tillering and maturity stages when fields were left without irrigation or drainage, the paddy soil remained moist or saturated throughout the growth periods. After the rice harvest, there was an approximately 7-month fallow period during which no irrigation or weeding was carried out.

### 2.3. Rice Growth Indexes Measurement

According to the actual growth conditions of the rice in the experimental plots, the following growth stages were defined after transplanting: 0–20 days as the Tillering Stage (TS), 20–36 days as the Jointing Stage (JS), 36–70 days as the Heading Stage (HS), 70–90 days as the Filling Stage (FS), and 90–115 days as the Maturity Stage (MS). At the rice TS, JS, HS, FS, and MS, the leaf area index (LAI) was measured using a leaf area meter (LAI-2200C, LI-COR, Lincoln, NE, USA), the SPAD was measured using a chlorophyll meter (502 Plus, KONICA MINOLTA, Japan), and the plant height was measured using a steel ruler with an accuracy of 1.0 mm.

### 2.4. Soil and Plant Sampling and Analysis

From 2019 to 2022, during each rice growing stage, soil samples were randomly collected from each experimental plot at depths of 0–10 and 10–20 cm using a soil auger, mixed, and then frozen at −18 °C for the determination of soil nitrate nitrogen and ammonium nitrogen. After the rice harvest in 2024, five soil samples were randomly collected from the 0–10 cm and 10–20 cm soil layers using a soil auger, following the five-point sampling method. The samples were then mixed, air-dried, ground, and sieved before their chemical properties, including their organic matter content, were analyzed.

According to the description in [20], NO_3_^−^-N (NN) was determined by ultraviolet and visible spectrophotometry; NH_4_^+^-N (AN) was determined by the indigo phenol blue colorimetric method; soil organic matter (SOM) was determined by the potassium dichromate volumetric method; total phosphorus (TP) was determined by the HClO_4_-H_2_SO_4_ method; alkaline hydrolyzable nitrogen (AHN) was determined by the alkaline diffusion method; available phosphorus (AP) was determined by the 0.5 mol·L^−1^ NaHCO_3_ method; available potassium (AK) was determined by the 1.0 mol·L^−1^ NH_4_OAc-flame photometric method; and soil pH was measured by the potentiometric method (a 1:2.5 soil/water ratio was used for testing; the soil and water were mixed, shaken, and left to stand before analysis). Soil total nitrogen (TN) was determined using a continuous flow analyzer (Systea Flowsys III) with the Kjeldahl digestion-indophenol blue colorimetric method according to the protocol described in [21]. Total potassium (TK) was determined by inductively coupled plasma mass spectrometry (ICP-MS) following microwave digestion, as described in [22].

At rice maturity stage each year, a 0.5 m border was excluded from each plot, and five rice hills were selected using a combined five-point sampling and localized random approach. These samples were transported to the laboratory for manual separation of grains and straw to determine yield components (including seed setting rate, 1000-grain weight, etc.) and grain moisture content. All remaining plants in the field were completely harvested by hand, threshed, dried, and weighed for yield calculation.

During the full bloom period of milk vetch, 1 m × 1 m quadrats with uniform growth were selected for plant sampling. The fresh weight of the samples was measured, then subjected to a pre-drying process at 105 °C for 30 min to kill green tissue, which was followed by drying at 70 °C until a constant weight was reached to determine biomass.

### 2.5. Statistical Analysis

All data were tested for normal distribution using SPSS 25.0 software (IBM, Armonk, NY, USA), which was followed by one-way ANOVA to determine differences in the rice yield and its components, tillering, and topsoil fertility among different treatments, with statistical significance set at *p* < 0.05. Pearson correlation analysis was used to determine the relationships among rice yield, growth parameters, and soil fertility. Linear regression analysis was performed to quantify the effects of yield components, and graphs of the results were generated using Origin 2024 software (OriginLab, Northampton, MA, USA).

Structural equation modeling (SEM) was performed using Amos 24 (IBM, USA) to evaluate the direct and indirect effects of fertilization on soil fertility, rice growth, and yield, and graphs of the results were prepared using PowerPoint 2022 (Microsoft, Redmond, WA, USA). Prior to modeling, the data underwent normality testing, necessary dimensionality reduction, and standardization using SPSS 25.0 software (IBM, USA). Missing values were handled through mean imputation. Both models incorporated 378 data points.

## 3. Results

### 3.1. Rice Yield and Its Components

**Figure 2:**
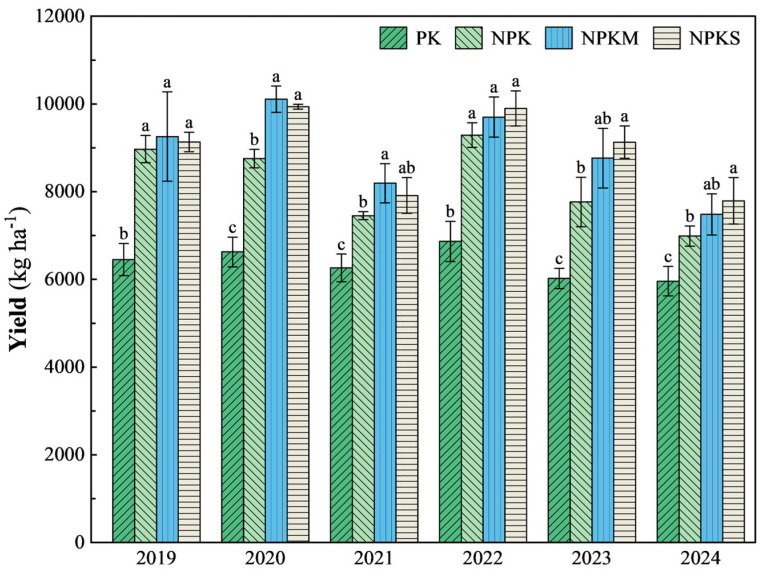
Rice yield of four different treatments from 2019 to 2024. Note: vertical bars represent the standard deviation, SD, of the mean (*n* = 3); different letters on the SD bar indicate significant differences between different treatment groups (*p* < 0.05).

Straw return and winter green manure have a significant positive impact on the yield of rice (Figure 2). Compared to the NPK and PK treatments, the six-year average yield increases of NPKS and NPKM were 9.52% and 40.75%, and 8.83% and 39.88%, respectively. The six-year average yields of the NPKS, NPKM, NPK, and PK treatments were 8966.65, 8917.04, 8202.90, and 6363.68 kg ha^−1^, respectively. The NPKM treatment achieved the highest yield from 2019 to 2021, peaking at 10,106.54 kg ha^−1^ in 2020, which was 15.5% and 52.5% higher than those of NPK and PK, respectively. Since 2022, the NPKS treatment has shown a consistent trend towards slightly higher mean yields than NPKM, although these differences have not been statistically significant. Notably, in 2023, the NPKS treatment achieved its highest yield, with an increase of 17.5% over NPK and 51.6% over PK.

The compositions of the rice yields under different treatments from 2019 to 2024 are shown in Table 1. NPKS and NPKM contribute to increases in effective panicles and the seed setting rate of rice, exhibiting multi-year average increases of 6.78%~32.66% and 1.03%~3.37% compared to the PK and NPK treatments. The annual average number of effective panicles in the NPKS treatment reached 286 × 10^4^ ha^−1^, which was the most significant improvement. However, there were no significant differences in the number of grains per panicle and the 1000-grain weight among the four treatments.

### 3.2. Rice Growth Index Measurement

Overall, the SPAD of rice under the four treatments showed a gradually decreasing trend from the TS to the MS; the LAI and plant height showed a gradually increasing trend from the TS to the MS, with the fastest growth occurring from the TS to the HS.

**Figure 3:**
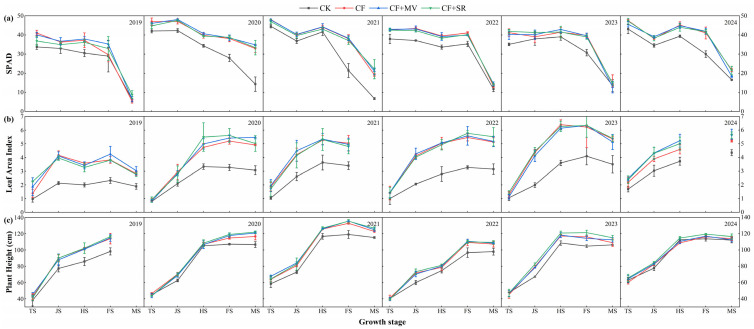
Rice SPAD value (**a**), leaf area index (**b**), and plant height (**c**) under different treatments from 2019 to 2024. Note: TS (Tillering Stage), JS (Jointing Stage), HS (Heading Stage), FS (Filling Stage), MS (Maturity Stage).

The NPKS and NPKM treatments improved the SPAD, LAI, and plant height of the rice, which were better than those of the NPK treatments, but without significant differences, and were significantly higher than those of the PK treatments (Figure 3). The NPKS treatment had the highest SPAD, LAI, and plant height, and the six-year average values of the three indicators were ranked as NPKS > NPKM > NPK > PK. Compared to the NPKM, NPK, and PK treatments, NPKS exhibited increases of 5.43%, 10.31%, and 16.81% in SPAD, respectively; increases of 15.80%, 32.04%, and 51.30% in LAI, respectively; and increases of 2.00%, 3.75%, and 6.61% in plant height, respectively.

NPKS and NPKM contribute to the tillering of rice, and the lack of nitrogen fertilizer significantly reduces the number of tillers throughout the entire growth period of rice (Table 2). The rice tillering increased rapidly from the TS to the JS and then gradually decreased. The NPKM and NPKS treatments maintained a high level throughout the entire growth period, especially NPKS, which showed a stable advantage in the MS, increasing by 18.62%~45.00%, 2.99%~13.64%, and 1.18%~5.95% compared to the PK, NPK, and NPKM treatments over five years, respectively. The five-year average of the NPKM and NPKS treatments increased by 10.32% and 22.83% and by 21.31% and 35.05%, respectively, compared to the NPK and PK treatments.

### 3.3. Properties of Topsoil After 2024 Harvest

The different management practices had varying degrees of impact on the fertility of the paddy soil in the topsoil (Table 3), with the 0–10 cm soil layer showing a more pronounced response than the 10–20 cm layer. The content of each nutrient element decreased with increasing depth. Overall, the NPKM and NPKS treatments exhibited higher values for multiple indicators than for the PK and NPK treatments which used only chemical fertilizers.

Management practices and the number of years of cultivation influence the SOM content. Straw return, winter green manure, and nitrogen fertilizer application are all beneficial for accumulating soil organic matter in the topsoil. The average SOM content in the 0–20 cm soil layer for the PK, NPK, NPKM, and NPKS treatments increased by 7.05%, 10.02%, 15.69%, and 17.02%, respectively, compared to the initial value of 44.56 g kg^−1^ (0–20 cm) at the start of the experiment. The NPKS treatment significantly increased the SOM content in the 0–10 cm soil layer compared to the NPK and PK treatments (increasing by 8.32% and 12.34%, respectively), reaching 64.14 g kg^−1^. No significant differences were observed among the four treatments in the 10–20 cm soil layer.

NPKS and NPKM increased the TN, TP, AHN, and AP content in the topsoil, following the order NPKS > NPKM > NPK > PK. The TN content of the NPKS treatment in the 0–10 cm soil layer was significantly higher than that of the PK and NPK treatments, with increases of 32.49%, 16.59%, and 9.27% compared to the PK, NPK and NPKM treatments, respectively. No significant differences were observed between the four treatments in the 10–20 cm soil layer. There were no significant differences in the TP and AHN content among the four treatments. For the AP content, that of NPKS was significantly higher than the that of other treatments in the 0–20 cm layer, increasing by 26.41%, 21.87%, and 12.41% compared to the PK, NPK, and NPKM treatments, respectively.

The NPK treatment had the lowest TK and AK contents. The average TK content of NPKM in the 0–20 cm layer was the highest (15.22 g kg^−1^), but no significant difference existed between treatments. The AK content of the NPKS treatment was significantly higher than that of the other treatments, increasing by 39.07%, 47.38%, and 32.11% compared to the PK, NPK, and NPKM treatments, respectively.

Different pH values were identified among these treatments. The soil pH in the 0–10 and 10–20 cm layers was in the order NPKM > PK > NPK > NPKS, with all having a higher pH in 10–20 cm than the 0–10 cm layer. It was found that long-term straw return and the application of chemical nitrogen fertilizer can lead to soil acidification. Compared to the PK and NPK treatments, the NPKS treatment decreased the soil pH by 0.07 and 0.04 units, respectively. The NPK treatment resulted in a slight decrease in the soil pH (0–20 cm) compared to the PK treatment, but the difference was insignificant. Green manure application increased the soil pH, with NPKM increasing the soil pH by 0.17 and 0.20 units compared to PK and NPK, respectively.

### 3.4. Dynamics of NN and AN in Topsoil During the Rice Growth Period

**Figure 4:**
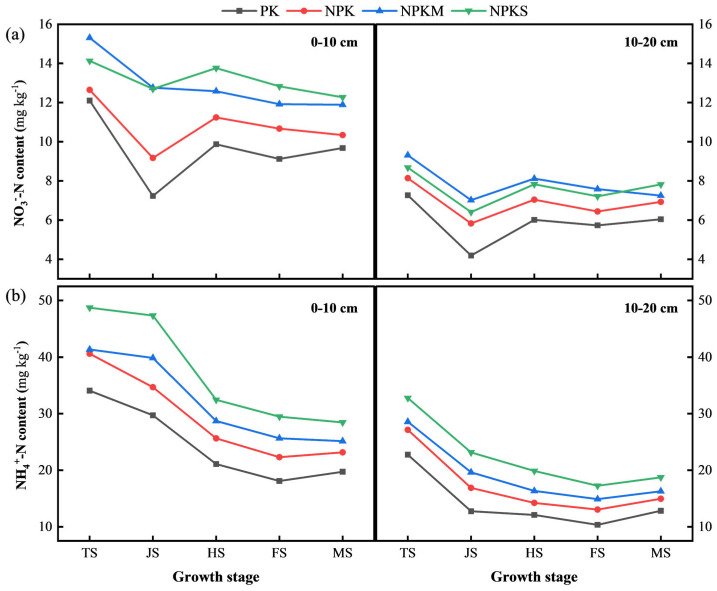
Changes in average NO_3_^−^-N (**a**) and NH_4_^+^-N (**b**) contents in topsoil during rice growth period from 2019 to 2022. Note: TS (Tillering Stage), JS (Jointing Stage), HS (Heading Stage), FS (Filling Stage), MS (Maturity Stage).

The NN and AN content of the topsoil is influenced by fertilization, rice growth, and depth (Figure 4). NPKS and NPKM significantly increased the mineral nitrogen content, with the order being NPKS > NPKM > NPK > PK. The NN and AN content in the 0–10 cm soil layer was significantly higher than in the 10–20 cm layer. With the application of basal fertilizer, the NN and AN content of all four treatments was relatively high, peaking during the TS, gradually decreasing with rice growth, and stabilizing after the FS.

The rate of decrease in NN content for NPKS and NPKM was significantly lower than for the PK and NPK treatments (Figure 4a). Specifically, from the TS to the JS in the 0–10 cm soil layer, the NN content decreased by 40.25%, 27.51%, 16.66%, and 10.12% for PK, NPK, NPKM, and NPKS, respectively. NPKS and NPKM maintained relatively high NN contents even at the MS.

The AN content of the NPKS treatment remained the highest throughout the entire growth stage (Figure 4b), especially in the 0–10 cm soil layer during the TS, reaching 48.73 mg kg^−1^, which was 17.9%, 23.9%, and 43.1% higher than that of the NPKM, NPK, and PK treatments, respectively.

### 3.5. Comprehensive Analysis of Rice Yield and Its Components, Growth Index, and Topsoil Properties

**Figure 5:**
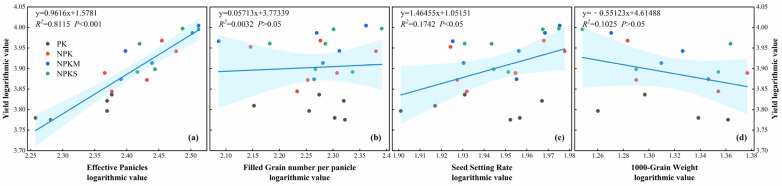
Linear regression analysis of rice yield and its components on a logarithmic scale: (**a**) effective panicles, (**b**) filled grain number per panicle, (**c**) seed setting rate, (**d**) 1000-grain weight.

The further linear regression analysis on a logarithmic scale showed that effective panicles (Figure 5a) had a highly significant positive effect on the yield (*p* < 0.001) and the seed setting rate (Figure 5c) had a significant positive effect on the yield (*p* < 0.05), while the filled grain number per panicle and 1000-grain weight (Figure 5b,d) had no significant effects on the yield (*p* > 0.05).

**Figure 6:**
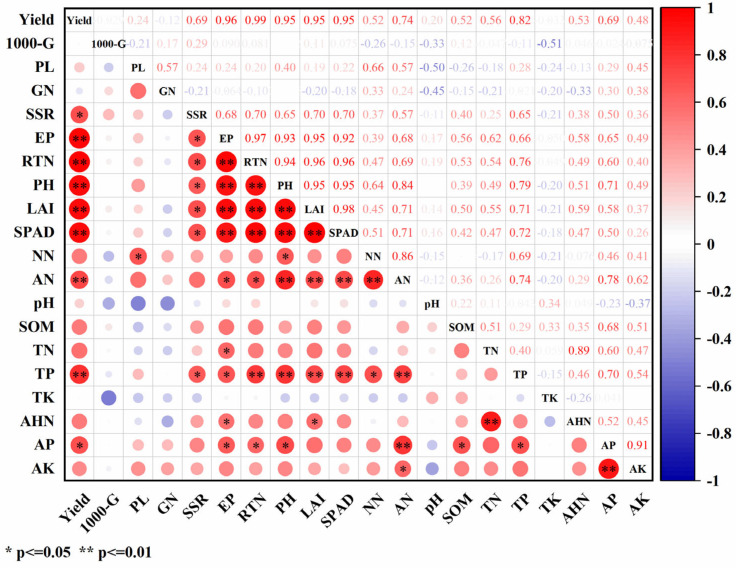
Correlation analysis of rice yield, growth, and 0–20 cm soil fertility. Note: 1000-G, 1000-grain weight; PL, panicle length; GN, grain number of rice; SSR, seed setting rate; EP, effective panicles; RTN, rice tiller number; PH, paddy height; LAI, leaf area index; NN, NO_3_^−^-N; AN, NH_4_^+^-N; SOM, soil organic matter; TN, soil total nitrogen; TP, soil total phosphorus; TK, soil total potassium; AHN, soil alkali hydrolyzable nitrogen; AP, soil available phosphorus; AK, soil available potassium.

Pearson correlation analysis (Figure 6) showed that the rice yield (RY) was highly positively correlated with rice growth indexes (effective panicles: EP, rice tiller number: RTN, plant height: PH, LAI, SPAD) and the soil fertility (AN, TP) (*p* ≤ 0.01), and was positively correlated with the seed setting rate (SSR) and AP (*p* ≤ 0.05). The soil fertility (AN, TP) was significantly positively correlated with rice growth indices (EP, RTN, PH, LAI, SPAD) (*p* ≤ 0.05 or *p* ≤ 0.01). Significant or highly significant positive correlations (*p* ≤ 0.05 or *p* ≤ 0.01) also exist among rice growth indicators such as the EP, RTN, PH, LAI, and SPAD.

**Figure 7:**
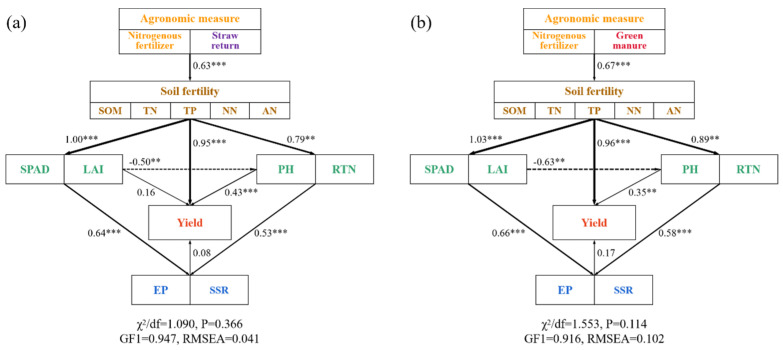
Structural equation model of straw returning (**a**), winter green manure (**b**), nitrogen fertilizer, soil fertility, and rice growth index on yield. Note: SSR, seed setting rate; EP, effective panicles; RTN, rice tiller number; PH, paddy height; LAI, leaf area index; SOM, soil organic matter; TN, soil total nitrogen; TP, soil total phosphorus; NN, NO_3_^−^-N; AN, NH_4_^+^-N. Solid and dashed arrows indicate positive and negative effects, respectively. Stars denote significance at *p* < 0.01 and *p* < 0.001 probability levels (** and ***, respectively).

In addition to Pearson correlation analysis, structural equation modeling (SEM) was used to analyze the direct and indirect effects of nitrogen fertilizer combined with straw return (Figure 7a) and winter green manure (Figure 7b) on soil fertility and rice growth indicators and the yield. The SEM results also confirmed our hypothesis. Both straw return + N fertilizer and winter green manure + N fertilizer directly increased the soil fertility, including the SOM, TN, TP, NN, and AN, indirectly promoting increases in the rice yield. The soil fertility improvement directly enhanced the SPAD, LAI, PH (plant height) and tillering, promoted rice growth, and indirectly improved the effective panicles and seed setting rate. The rice yield was affected by multiple factors, and the improvement in soil fertility was also the most significant influencing factor on rice yield, exhibiting correlations of 0.95 and 0.96, respectively (Figure 7a,b).

## 4. Discussion

### 4.1. Response of Soil Organic Matter Content to Straw Returning to the Field and Winter Green Manure

Straw return and winter green manure incorporation are essential for increasing soil organic matter content. Our research found that the SOM content increased by 8.07% and 5.16% with the NPKS and NPKM treatments, respectively, compared to the NPK treatment. This is consistent with the findings of Wang et al. in 2018 [23] and Wang et al. in 2025 [24]. This was because the straw and green manure acted as direct inputs of exogenous organic matter, replenishing the soil carbon pool [25] and also increasing carbon input from subsequent rice stubble and root biomass [26,27]. The PK and NPK treatments also showed slight increases in the SOM content compared to the initial levels. This may be attributed to the single rice cropping system, which included a 7-month undisturbed fallow period that minimized the SOM loss [28,29], and the non-use of herbicides, which allowed weed growth (e.g., *Polypogon* spp.) that was incorporated into the soil during pre-planting tillage, collectively contributing to modest SOM accumulation.

However, in our study, the average annual growth rate of the SOM in NPKS and NPKM over six years compared with the NPK treatment was 1.06% and 0.86%, respectively, much lower than the observation results in more barren areas. In the arid region of Ningxia, China, straw return increased the average annual organic carbon by 5.26% compared to conventional tillage over six years [23], while in the red soil area of Hunan Province, China, milk vetch incorporation increased the average annual organic carbon by 3.25% compared to conventional tillage over four years [30]. This difference may be because our experimental site’s soil organic carbon level was already near saturation [31]. According to the soil carbon saturation theory, the further the soil carbon is from saturation, the greater the potential for exogenous carbon addition to increase SOC storage [18]. A meta-analysis also indicated a significant negative linear relationship between the SOC response ratio and the initial carbon concentration under straw return conditions, and that the soil C would saturate 12 years after straw C input [32].

The substantial input of organic materials did not significantly increase the soil organic matter content, which indicated that much carbon was lost. The research conducted by Liu et al. [32] in 2014 pointed out that the soil might reach carbon saturation 12 years after the input of straw, and that the emissions of CH_4_ and CO_2_ would increase. A global meta-analysis indicated that green manure combined with inorganic fertilizer increased the greenhouse gas emissions during the rice growing season [33]. Therefore, when soil organic matter reaches a high level (close to carbon saturation), we may need to reduce the amount of organic material returned to the soil to achieve a balance between maintaining soil organic carbon levels and reducing negative impacts on the environment [34]. Whether our study has reached carbon saturation needs further investigation, and we will use longer cycles and the monitoring of greenhouse gases to assess soil carbon sequestration potential accurately.

### 4.2. The Different Effects of Straw and Winter Green Manure on Topsoil Properties

Our research results indicate that straw returning to the field and winter green manure have improved the fertility of the top rice-planting soil (Table 3, Figure 4). Both straw and green manure increased the TN, NN, and AN content in the topsoil, but the mechanisms and dynamic changes differed. Milk vetch can be used as a leguminous green manure which inputs a large amount of nitrogen into the soil through symbiotic nitrogen fixation by rhizobia, increasing the TN content [35]. After incorporation, rapid mineralization releases amino sugars, increasing the NN and AN [36]. The NN content in the topsoil under the NPKM treatment during the TS was at its highest level (Figure 4a). This was due not only to the application of basal fertilizer but also to the lower C/N ratio of milk vetch and the increase in spring temperature and moisture content, which accelerated residue decomposition and resulted in the release of a large amount of nitrogen. In contrast, due to its high C/N ratio, straw return can cause microbial nitrogen immobilization in the early stages of decomposition, which can lead to a short-term decrease in the available nitrogen [37]. After long-term decomposition, mineralization can significantly increase the total nitrogen content [38]. Our results demonstrated that, after six years of straw return, the high C/N straw substrate provided substantial nutrients to the soil through prolonged decomposition. The NPKS treatment resulted in the highest topsoil SOM and TN contents (Table 3), which led to a lower C/N ratio compared to the other three treatments (Table 3).

The soil total phosphorus and available phosphorus content also responded positively to straw and green manure incorporation (Table 3). In the NPKM treatment, it was observed that the root system of milk vetch directly promotes nutrient activation through rhizosphere acidification and citric acid secretion, effectively dissolving the occluded phosphorus pool. The rhizosphere effect accelerated the transformation of unavailable P to available P, increasing the soil phosphorus content [17,39]. On the other hand, straw return promotes soil aggregate formation and provides substrates for soil enzymes, enhancing their activity [40,41] and increasing the mineralization rate of organic phosphorus and phosphorus content [42,43].

Previous studies have yielded inconsistent conclusions regarding the effect of winter green manure and straw return treatments on soil potassium content. Many studies have found this effect positive [25,44,45]. At the same time, a trial conducted in the subtropical double-cropping rice area of China indicated that the incorporation of milk vetch and straw reduced (7.12%) and increased (156.75%) the soil available potassium content, respectively, compared to no incorporation [46]. In this study, the NPKM treatment had the highest total potassium content (11.62% higher than NPK), the NPKS treatment had the highest available potassium content (47.52% higher than NPK), and the NPK treatment had the lowest. A field experiment in southern China reported similar findings: straw return in early rice increased the available potassium content by 61.57% compared to chemical fertilizer application alone [25]. This may be because nitrogen deficiency under the PK treatment limits the growth of rice plants [5], resulting in lower potassium uptake and the decomposition of straw and green manure produces organic acids that promote the activation and release of mineral potassium. Furthermore, the potassium in straw primarily exists in ionic form, which is readily hydrolyzed after return. The humification process of straw also reduces the potassium fixation on clay minerals and facilitates the conversion of slow-release potassium to available potassium [47]. However, the NPK treatment lacks exogenous organic matter to supplement potassium.

However, it is worth noting that the NPKM and NPKS treatments have drastically different effects on soil pH. The alkaline metabolites (e.g., betaine) released during the incorporation and decomposition of milk vetch increase the soil pH [48,49]. For instance, Wang et al. [49] demonstrated that incorporating 12 g kg^−1^ milk vetch could provide approximately 10 mmol of alkalinity to the soil [50]. Conversely, the organic acids produced during straw decomposition decrease the soil pH [51,52]. Kumari et al. [52] showed, in an incubation experiment, that rice straw produced 0.46 µmol g^−1^ straw of citric acid and 0.84 µmol g^−1^ straw of oxalic acid after 15 days of decomposition [53]. In the long term, the risk of soil acidification caused by straw incorporation must be considered.

### 4.3. Effects of Straw Return and Winter Green Manure on Rice Growth and Yield

The results of this study indicate that the rice yields under the NPKS and NPKM treatments were significantly higher than those under the PK and NPK treatments (Figure 2), which is consistent with previous research conclusions on the yield-increasing effects of green manure and straw return in rice [54,55]. Notably, the temporal effects of different agronomic measures in our study showed significant differences: in the first three years of the experiment (2019–2021), the NPKM treatment had the most significant yield advantage, while in the subsequent observation period (2022–2024), the NPKS treatment became the highest-yielding treatment group. This difference may be closely related to the hydrological characteristics of the study area and the unique decomposition mechanisms after straw return.

Because the study area is located in the densely networked Jianghan Plain, which has a shallow groundwater table, the surface soil moisture content remained consistently high during the winter fallow period, limiting milk vetch growth [56]. Despite water regulation through field ditch drainage systems, the rapid expansion of the rice-crayfish cultivation area further raised the groundwater table in the study area (the groundwater depth during the non-rice growth period decreased from approximately 0.7 m in 2019 to approximately 0.3 m in 2023). This change in the hydrological environment directly led to a significant downward trend in the milk vetch biomass (from 3.38 t ha^−1^ in 2022 to 2.92 t ha^−1^ in 2023, Table S1), thereby reducing the nutrient input of the green manure system.

Linear regression analysis showed that the effective panicles and seed setting rate were the most influential yield components (Figure 5). The Tillering stage and Filling stage emerged as the key periods that determined the panicle number and seed setting rate, respectively. The tillering was significantly influenced by the nitrogen availability [57], and the NPKS and NPKM treatments brought more mineral nitrogen to the topsoil during the Tillering stage (Figure 4) and thereby promoted tillering (Table 2). Improvements in rice growth and development, including in the SPAD, LAI, plant height, and tillering of the rice, significantly positively impacted the rice yield and its components (Figure 6 and Figure 7). The SPAD value is widely used to estimate the total chlorophyll content [58], the LAI characterizes the density of the leaves and canopy structure [59], and the number of tillers is a key determinant of the number of rice panicles [60]. In this study, the application of straw and green manure improved the SPAD, LAI, plant height, and tillering of rice, which is consistent with previous studies [61,62]. However, in our study, the rice tiller number decreased as the plants matured, which might be attributed to sampling errors. Correlation analysis indicated (Figure 6) that the TP content had a significant positive correlation with the seed setting rate (*p* ≤ 0.05), and the increased TP content in the topsoil under the NPKS and NPKM treatments might have led to the improved seed setting rate, which aligns with previous findings [63]. Correlation analysis and SEM results showed (Figure 6 and Figure 7) that, with the input of straw and green manure, the improvements in the soil fertility and rice growth and development were strongly positively correlated, which was conducive to increasing the rice yield. The straw return system exhibited unique nutrient dynamic characteristics compared to the green manure treatment. In the initial stage (2019), the rice SPAD and LAI of the NPKS treatment were lower than those of the other treatments (Figure 3), which is related to the nitrogen competition mechanism between microorganisms and crops during the decomposition of high-C/N-ratio straw [37,64].

## 5. Conclusions

This study systematically evaluated the long-term effects of straw and green manure return on high-fertility paddy fields in southern China through a six-year field experiment. The results showed that straw and green manure return significantly increased rice yields compared to conventional fertilization, with an average increase of 9.52% and 8.83% over six years, respectively. The green manure treatment had the highest yield in the first three years (2019–2021), while the straw return showed a more sustained yield advantage from the fourth year (2022). The most important finding of this study is that, in the lowland river network area of subtropical plains with high fertility accumulation, returning straw and green manure as organic materials to the field for six consecutive years can still increase the content of soil organic matter (at a rate lower than that in areas with soil fertility) and increase the content of nutrients such as nitrogen, phosphorus, and potassium. However, straw slightly leads to soil acidification. The two organic management measures improved the soil fertility and key rice growth indicators (tiller number, SPAD, LAI, and plant height), thereby increasing the yield. Both can be used as effective agronomic measures for the efficient utilization of organic resources and the maintenance of food security in subtropical high-fertility rice areas. However, subsequent research needs to focus on the long-term threshold effect of organic-inorganic fertilization and its impact on greenhouse gas emissions.

## Figures and Tables

**Table 1:** The rice yield components of four different treatments during 2019–2024.

Year	Treatment	Effective Panicles (10^4^·ha^−1^)	Filled Grain Number per Panicle	Seed Setting Rate (%)	1000-Grain Weight (g)
2019	PK	-	141 ± 21 a	82.6 ± 5.2 a	-
	NPK	-	140 ± 33 a	84.0 ± 5.6 a	-
	NPKM	-	122 ± 47 a	84.2 ± 7.2 a	-
	NPKS	-	152 ± 15 a	85.1 ± 2.4 a	-
2020	PK	234 ± 26 b	209 ± 32 a	92.7 ± 3.9 a	-
	NPK	300 ± 27 ab	240 ± 13 a	95.0 ± 3.3 a	-
	NPKM	325 ± 32 a	230 ± 2 a	94.5 ± 3.8 a	-
	NPKS	307 ± 34 ab	246 ± 17 a	94.4 ± 3.1 a	-
2021	PK	234 ± 15 b	180 ± 17 a	79.8 ± 6.9 a	18.2 ± 0.1 a
	NPK	270 ± 12 ab	179 ± 40 a	84.6 ± 5.7 a	19.5 ± 0.6 a
	NPKM	275 ± 24 ab	191 ± 39 a	85.2 ± 4.0 a	20.4 ± 2.1 a
	NPKS	278 ± 24 a	185 ± 13 a	88.0 ± 5.3 a	19.5 ± 0.6 a
2022	PK	238 ± 12 b	188 ± 8 a	85.1 ± 4.4 b	19.8 ± 3.4 a
	NPK	285 ± 6 ab	189 ± 18 a	92.9 ± 1.6 a	19.2 ± 0.3 a
	NPKM	318 ± 35 a	186 ± 11 a	93.0 ± 1.7 a	18.6 ± 0.4 a
	NPKS	323 ± 25 a	200 ± 21 a	92.8 ± 2.2 a	17.7 ± 1.5 a
2023	PK	181 ± 28 b	201 ± 17 a	90.5 ± 0.7 a	21.8 ± 0.5 a
	NPK	232 ± 24 ab	203 ± 22 a	90.1 ± 2.0 a	23.8 ± 1.5 a
	NPKM	250 ± 16 ab	205 ± 17 a	90.6 ± 1.9 a	21.2 ± 0.5 a
	NPKS	263 ± 62 a	193 ± 15 a	87.8 ± 3.5 a	23.1 ± 2.4 a
2024	PK	191 ± 29 b	210 ± 31 a	89.6 ± 2.8 ab	23.0 ± 1.3 a
	NPK	238 ± 40 ab	171 ± 33 a	85.5 ± 2.5 b	22.6 ± 0.8 a
	NPKM	246 ± 28 ab	184 ± 14 a	90.4 ± 1.8 a	22.2 ± 0.1 a
	NPKS	261 ± 8 a	217 ± 18 a	89.4 ± 1.2 ab	22.6 ± 1.1 a

Note: Different letters within the same year indicate significant differences (*p* < 0.05).

**Table 2:** The effects of four different treatments on the tiller number during 2020–2024.

Year	Treatments	Tillering Stage	Jointing Stage	Heading Stage	Filling Stage	Maturity Stage
2020	PK	11.9 ± 0.8 b	-	12.1 ± 1.3 b	-	-
	NPK	17.8 ± 2.1 a	-	14.3 ± 1.1 ab	-	-
	NPKM	18.8 ± 0.5 a	-	16.7 ± 1.6 a	-	-
	NPKS	17.5 ± 1.3 a	-	15.8 ± 1.7 a	-	-
2021	PK	9.3 ± 2.3 b	-	10.7 ± 0.4 b	8.9 ± 1.1 b	9.7 ± 0.6 b
	NPK	12.1 ± 2.4 ab	-	15.7 ± 1.0 ab	12.7 ± 0.3 a	11.1 ± 0.5 ab
	NPKM	12.6 ± 1.6 ab	-	14.2 ± 3.3 a	12.5 ± 1.4 a	11.3 ± 1.0 ab
	NPKS	13.8 ± 1.1 a	-	15.4 ± 0.2 a	12.3 ± 2.6 a	11.5 ± 1.0 a
2022	PK	13.1 ± 2.5 a	15.9 ± 1.6 b		12.5 ± 2.0 b	12.3 ± 0.6 b
	NPK	13.7 ± 4.4 a	25.6 ± 2.4 ab		17.2 ± 2.7 a	14.7 ± 0.3 ab
	NPKM	15.5 ± 1.6 a	28.9 ± 2.6 a		17.9 ± 1.3 a	16.4 ± 1.8 a
	NPKS	14.6 ± 2.3 a	23.8 ± 8.1 ab		18.0 ± 2.1 a	16.7 ± 1.3 a
2023	PK	7.5 ± 0.8 a		11.5 ± 1.0 b	10.6 ± 0.9 b	9.3 ± 1.4 b
	NPK	10.8 ± 2.3 a		19.2 ± 2.6 a	15.3 ± 0.8 a	11.9 ± 1.2 ab
	NPKM	9.7 ± 3.2 a		18.4 ± 4.4 a	16.1 ± 1.6 a	12.9 ± 0.8 ab
	NPKS	11.5 ± 1.6 a		20.1 ± 2.6 a	16.7 ± 1.2 a	13.5 ± 3.2 a
2024	PK	9.6 ± 3.1 a	11.9 ± 2.5 b	11.0 ± 1.4 a	9.1 ± 1.1 b	9.9 ± 1.5 b
	NPK	11.7 ± 1.1 a	14.1 ± 3.2 ab	15.3 ± 2.3 a	13.7 ± 0.5 a	12.3 ± 2.0 ab
	NPKM	15.9 ± 6.0 a	18.1 ± 1.9 a	14.7 ± 2.3 a	13.7 ± 1.1 a	12.7 ± 1.5 ab
	NPKS	16.3 ± 1.2 a	17.4 ± 2.3 a	14.9 ± 3.3 a	14.0 ± 1.8 a	13.5 ± 0.4 a

Note: Different letters in the same column indicate significant differences among treatments (*p* < 0.05).

**Table 3:** Soil nutrient status of topsoil after rice harvest in 2024.

Treatments	Depth (cm)	pH (-)	SOM (g kg^−1^)	TN (g kg^−1^)	TP (g kg^−1^)	TK (g kg^−1^)	AHN (mg kg^−1^)	AP (mg kg^−1^)	AK (mg kg^−1^)	C/N (-)
PK	0–10	7.41 ± 0.05 b	57.1 ± 6.6 c	1.82 ± 0.16 b	0.93 ± 0.06 a	15.3 ± 3.9 a	106 ± 8 a	9.4 ± 0.2 c	91.0 ± 5.7 c	18.3 ± 3.0 a
NPK		7.38 ± 0.05 bc	59.2 ± 4.4 bc	1.94 ± 0.29 b	1.05 ± 0.13 a	13.0 ± 1.6 a	110 ± 5 a	10.2 ± 1.5 bc	87.2 ± 4.2 c	17.8 ± 3.4 a
NPKM		7.59 ± 0.02 a	63.3 ± 1.8 ab	2.15 ± 0.31 ab	1.09 ± 0.28 a	15.2 ± 1.2 a	112 ± 11 a	10.9 ± 0.3 b	98.7 ± 1.5 b	17.3 ± 2.6 a
NPKS		7.34 ± 0.06 c	64.1 ± 4.0 a	2.33 ± 0.47 a	1.13 ± 0.09 a	13.7 ± 2.4 a	115 ± 7 a	12.5 ± 1.0 a	131 ± 4 a	16.8 ± 2.9 a
PK	10–20	7.47 ± 0.08 b	38.3 ± 1.8 a	1.12 ± 0.27 a	0.86 ± 0.18 a	14.9 ± 2.9 a	69.8 ± 6.0 a	8.6 ± 0.4 b	91.3 ± 8.1 bc	20.0 ± 2.3 a
NPK		7.45 ± 0.02 bc	38.8 ± 2.6 a	1.30 ± 0.29 a	0.98 ± 0.30 a	14.3 ± 1.3 a	70.6 ± 6.8 a	8.7 ± 0.6 b	84.6 ± 5.8 c	17.7 ± 2.6 a
NPKM		7.64 ± 0.02 a	39.8 ± 2.0 a	1.34 ± 0.09 a	1.03 ± 0.03 a	15.3 ± 2.4 a	72.6 ± 13.8 a	9.4 ± 1.0 b	92.9 ± 1.7 b	17.6 ± 2.1 a
NPKS		7.40 ± 0.07 c	40.1 ± 2.5 a	1.43 ± 0.29 a	1.06 ± 0.06 a	16.4 ± 1.6 a	74.1 ± 4.1 a	10.3 ± 1.1 a	122 ± 1 a	16.6 ± 1.9 a

Note: SOM, soil organic matter; TN, soil total nitrogen; TP, soil total phosphorus; TK, soil total potassium; AHN, soil alkali hydrolysable nitrogen; AP, soil available phosphorus; AK, soil available potassium. Different letters within the same depth indicate significant differences (*p* < 0.05).

## Data Availability

The original contributions presented in this study are included in the article/Supplementary Materials. Further inquiries can be directed to the corresponding author.

## Supplementary Materials

The following supporting information can be downloaded at: https://www.mdpi.com/article/10.3390/plants14131967/s1. Table S1: Biomass of milk vetch from 2022 to 2023.
