# Supplementary material for: Straw and Green Manure Return Can Improve Soil Fertility and Rice Yield in Long-Term Cultivation Paddy Fields with High Initial Organic Matter Content
Source: Plants (Basel). 2025 Jun 27;14(13):1967. doi: 10.3390/plants14131967 (PMC12251679; doi:10.3390/plants14131967)
Supplement: Supplementary file 1 [file plants-14-01967-s001.zip › plants-3645804-supplementary.pdf]

**Year**	**Milk Vetch Biomass** (t ha^{-1})
2022	3.38
2023	2.92

Supplementary Table S1: Biomass of milk vetch from 2022 to 2023.
